# Enhanced radiation use efficiency and grain filling rate as the main drivers of grain yield genetic gains in the CIMMYT elite spring wheat yield trial

**DOI:** 10.1038/s41598-024-60853-6

**Published:** 2024-05-14

**Authors:** Guillermo Gerard, Suchismita Mondal, Francisco Piñera-Chávez, Carolina Rivera-Amado, Gemma Molero, Jose Crossa, Julio Huerta-Espino, Govindan Velu, Hans Braun, Ravi Singh, Leonardo Crespo-Herrera

**Affiliations:** 1 International Maize and Wheat Improvement Center (CIMMYT), Carretera México- Veracruz Km. 45, El Batán, CP 56237 Texcoco, Mexico Mexico; 2 Plant Sciences and Plant Pathology Department, Montana State University, Bozeman, MT 59717 USA; 3 KWS Momont Recherche, 59246 Mons-en-Pévèle, Hauts-de-France France; 4 Colegio de Postgraduados, CP 56230 Montecillos, Mexico Mexico; 5 Campo ExperimentalValle de Mexico-INIFAP, Texcoco, Mexico Mexico

**Keywords:** Agricultural genetics, Plant breeding, Quantitative trait

## Abstract

Common wheat (*Triticum aestivum* L.) is a major staple food crop, providing a fifth of food calories and proteins to the world’s human population. Despite the impressive growth in global wheat production in recent decades, further increases in grain yield are required to meet future demands. Here we estimated genetic gain and genotype stability for grain yield (GY) and determined the trait associations that contributed uniquely or in combination to increased GY, through a retrospective analysis of top-performing genotypes selected from the elite spring wheat yield trial (ESWYT) evaluated internationally during a 14-year period (2003 to 2016). Fifty-six ESWYT genotypes and four checks were sown under optimally irrigated conditions in three phenotyping trials during three consecutive growing seasons (2018–2019 to 2020–2021) at Norman E. Borlaug Research Station, Ciudad Obregon, Mexico. The mean GY rose from 6.75 (24th ESWYT) to 7.87 t ha^−1^ (37th ESWYT), representing a cumulative increase of 1.12 t ha^−1^. The annual genetic gain for GY was estimated at 0.96% (65 kg ha^−1^ year^−1^) accompanied by a positive trend in genotype stability over time. The GY progress was mainly associated with increases in biomass (BM), grain filling rate (GFR), total radiation use efficiency (RUE_total), grain weight per spike (GWS), and reduction in days to heading (DTH), which together explained 95.5% of the GY variation. Regression lines over the years showed significant increases of 0.015 kg m^−2^ year^−1^ (*p* < 0.01), 0.074 g m^−2^ year^−1^ (*p* < 0.05), and 0.017 g MJ^−1^ year^−1^ (*p* < 0.001) for BM, GFR, and RUE_total, respectively. Grain weight per spike exhibited a positive but no significant trend (0.014 g year^−1^, *p* = 0.07), whereas a negative tendency for DTH was observed (− 0.43 days year^−1^, *p* < 0.001). Analysis of the top ten highest-yielding genotypes revealed differential GY-associated trait contributions, demonstrating that improved GY can be attained through different mechanisms and indicating that no single trait criterion is adopted by CIMMYT breeders for developing new superior lines. We conclude that CIMMYT’s Bread Wheat Breeding Program has continued to deliver adapted and more productive wheat genotypes to National partners worldwide, mainly driven by enhancing RUE_total and GFR and that future yield increases could be achieved by intercrossing genetically diverse top performer genotypes.

## Introduction

Bread wheat (*Triticum aestivum* L.) is one of the most important staple crops worldwide. It is grown on over 215 million hectares with a global production of over 735 million tons, providing a fifth of food calories and proteins to the world’s population^1^. Hence, it plays a crucial role in global food security and agri-food systems around the globe^2^. Despite the impressive growth in global wheat production in recent decades, it is still a challenge to meet the future wheat demand caused by the increase in global population, dietary shifts, and the detrimental effects of climate change^3^. Wheat production needs to increase using limited resources combined with climate change events and rising energy costs^4^. To face these challenges and continue to increase wheat productivity, continuous development and dissemination of nutritious and market-acceptable varieties that are climate resilient, disease resistant, and have higher and stable yields is necessary along with better agronomic practices, inputs management, and conducive agricultural policies.

The International Maize and Wheat Improvement Center (CIMMYT), since its establishment in 1966 from the success of the Green Revolution, has led the International Wheat Improvement Network (IWIN) that has continued to play an important role in developing higher-yielding, widely adapted, and stably performing wheat varieties in targeted countries of Asia, Africa, and Latin America as well as beyond. Nearly half of the wheat varieties grown around the world, and 70–80% of all varieties grown in South Asia, Central and West Asia, and North Africa, are derived from CIMMYT breeding research^5^. In these regions, the CIMMYT Bread Wheat Breeding Program targets nearly 60 million hectares (i.e., over one-quarter of the global wheat harvested area)^6^. Given the range and diversity of environments and to improve the selection efficiency as well as to address local needs, the CIMMYT Bread Wheat Breeding Program leverages yield phenotyping in diverse simulated environments at Ciudad Obregon together with National partners’ multi-location testing following the concept of mega-environments (MEs) and more recently target population of environments (TPEs)^7,8^. New elite lines bred each year are shared with nearly 250 partner institutions, both public and private, in about 70 countries worldwide through targeted IWIN yield trials and screening nurseries. Particularly, the Elite Spring Wheat Yield Trial (ESWYT) is the longest-running IWIN trial targeting optimally irrigated, high-yielding environments.

Quantifying GY genetic progress is essential in assessing breeding strategies as well as the overall performance of a breeding program^9^. The IWIN platform allows yearly evaluations of elite germplasm at several locations in various countries, and by including long-term checks in such trials, it is possible to evaluate the relative rate of GY progress throughout a determined period^10–12^. Previous studies using internationally distributed trial data have shown continuous genetic progress in CIMMYT’s ESWYT germplasm. An early study analyzing 16th–30th ESWYTs found a yield increase rate of 27.4 kg ha^−1^ year^−1^ (0.55%) relative to genotype Atilla in irrigated sites representing mega-environment 1 (ME1; optimal growth conditions environment)^10^. Meanwhile, more recent research using a factor analytic model for assessing the GE interaction reported genetic gains in the order of 102.7 kg ha^−1^ year^−1^ (1.63%) in the 27th to 34th ESWYT period when compared to the long-term CIMMYT check Atilla^11^. Although the estimation of yield progress using international trials data is widely accepted, another strategy is to assess the yield genetic gains per se by conducting well-managed on-station trials under high-yield potential and disease-free conditions for a set of elite germplasm developed/released during a defined time period.

Improving grain yield has always been a difficult task as it is a quantitative and complex trait determined by several agronomic and physiological features and strongly influenced by environmental and crop management factors^13^. Therefore, in addition to assessing the GY genetic progress, studying the changes in related traits is a critical step to determine their associations, identify yield-limiting factors, and especially plan effective approaches to steadily increase the GY genetic gains in breeding programs. Periodic evaluation of released cultivars for associated changes in physiological and agronomic traits and their relative contribution can provide a much-needed clue for future breeding strategies. Several studies have discussed yield components contributing to GY progress^3,9,14^. A major jump in GY gains was achieved during the Green Revolution with the introduction of dwarfing genes, which by reducing the size of vegetative plant organs resulted in better availability of assimilates to reproductive organs thereby leading to higher yields through an improved harvest index^15,16^. Further research has also shown that additional genetic progress in GY has been related to kernel number per unit of land area^17^, whereas more recent studies on CIMMYT germplasm suggested that the recent progress was mainly associated with flowering time, grain size, grain weight, and grain filling period^12,14,18^. Further improvements are expected to come through enhanced biomass production and radiation use efficiency by integrating modern high-throughput phenotyping and genomic tools together with a better understanding of physiological processes^19,20^, along with structural and agronomic adjustments for lodging tolerance^20–22^. Different theoretical estimates from the literature show that the radiation use efficiency of current wheat lines could be increased by up to 50% under favorable crop-growing conditions^23,24^, where even small increases in RUE and net photosynthesis rates could have a large impact on GY^25^.

The objectives of our study were (i) to estimate the annual GY genetic gain and changes in stability of superior performing ESWYT genotypes selected from 14 year period (2003 to 2016) by growing them in common trials under well-managed, optimally irrigated, and disease-free conditions; (ii) to determine the genetic changes that occurred for agronomic and physiological traits during the time period; and (iii) to determine the relative-traits associations that contributed uniquely or in combination to increase GY.

## Results

Changes in Grain yield plus 35 agronomic and physiological traits (see material and methods section) were assessed in 54 ESWYT elite lines (developed from 2003 to 2016) and 4 checks, grown under nine optimal conditions trials.

### Elite spring wheat yield trials grain yield genetic gains

**Figure 1:**
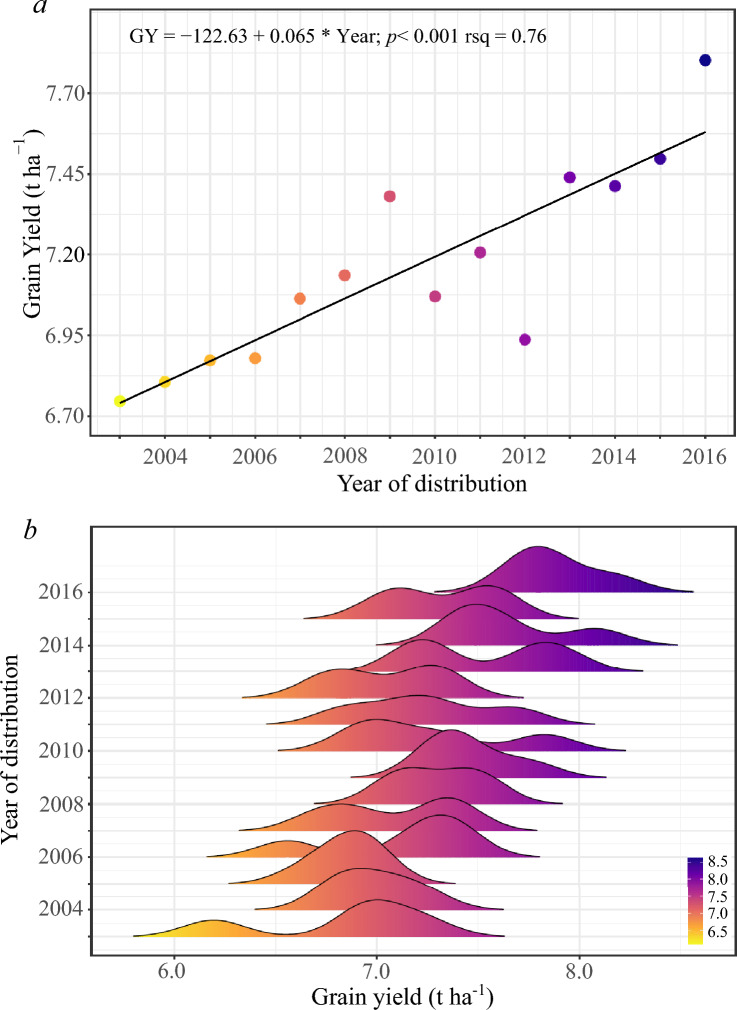
Grain yield genetic progress for top-performing CIMMYT’s international elite spring wheat yield trials (ESWYT) genotypes selected from trials distributed from 2003 to 2016 (24th to 37th ESWYT) and phenotyped under optimal conditions at Norman E. Borlaug Experiment Station from 2018 to 2021. (**a**) Average grain yield genetic gain (t ha^−1^) for the 14 years analyzed period; (**b**) grain yield density distribution of all ESWYT lines included in the trials.

**Figure 2:**
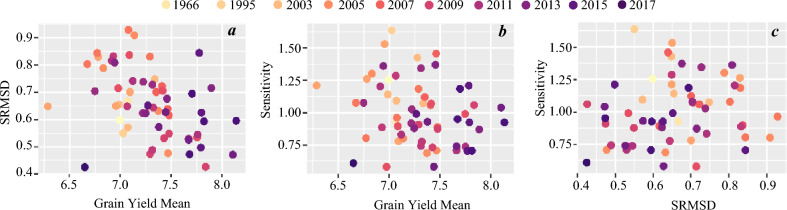
Finlay–Wilkinson stability analysis for 56 ESWYT elite lines plus four checks grain yield. (**a**) SRMSD (square root of the mean squared deviation) *vs.* grain yield mean; (**b**) Sensitivity *vs*. grain yield mean; and (**c**) Sensitivity *vs.* SRMSD.

Seven out of nine trials exhibiting significant GY correlations between them, as well as moderate to high GY heritability values (Table S3) (two trials with heritability < 0.15 were discarded from further analysis), were used to estimate GY genetic gains. The average GY across trials ranged from 6.05 (F5IR 19–20) to 9.08 (B5IR 18–19) t ha^−1^. Over the years of ESWYT distribution, the average GY ranged from 6.75 (24th ESWYT) to 7.87 t ha^−1^ (37th ESWYT), whereas the average GY of the checks across trials ranged between 6.14 and 7.83 t ha^−1^. Significant (*p* < 0.001) GY genetic progress was observed (Fig. 1). The annual rate of GY increase was 0.96%, which represents 65 kg ha^−1^ year^−1^ and a cumulative total of ~ 1.12 t ha^−1^ from a base of 6.75 t ha^−1^ (Fig. 1a). In addition, as shown in Fig. 1b the density plot created using the complete set of lines data also displays substantial GY increases over time. When the GY progress of the ESWYT lines included in the trials was calculated relative to the check average, the analysis again revealed genetic gains of around 0.96% (data not shown). According to the analysis of orthogonal polynomials, there was a significant linear trend of genetic gains per se and in terms of the checks (*p* < 0.001) with R^2^ of 0.76 and 0.77, respectively, while the quadratic response was found to be non-significant (*p* = 0.099), indicating no signs of genetic gains stagnation (Table 1). The genotype stability assessment reveals a positive trend toward greater stability (Fig. 2a–c). Thus, several of the most recently developed lines exhibit higher GY along with enhanced stability over the range of performed experiments (lower sensitivity and square root of the mean squared deviation).

**Table 1:** Orthogonal polynomials analysis to assess linearity versus non-linearity traits response.

Trait	Contrast	DF	Contrast SS	F value	Pr > F
GY	Linear effect	1	53.30	155.65	< 0.0001
	Quadratic effect	1	0.94	2.73	0.099
RUE_total	Linear effect	1	0.81	23.45	< 0.0001
	Quadratic effect	1	0.06	1.62	0.2052
BM	Linear effect	1	1,002,402.19	34.49	< 0.0001
	Quadratic effect	1	6505.20	0.22	0.6367
GFR	Linear effect	1	27.04	14.39	0.0002
	Quadratic effect	1	1.17	0.62	0.4311
GWS	Linear effect	1	0.63	23.52	< 0.0001
	Quadratic effect	1	0.01	0.5	0.4817
DH	Linear effect	1	3194.29	2463.28	< 0.0001
	Quadratic effect	1	22.94	17.69	< 0.0001

### Grain yield and associated traits

**Figure 3:**
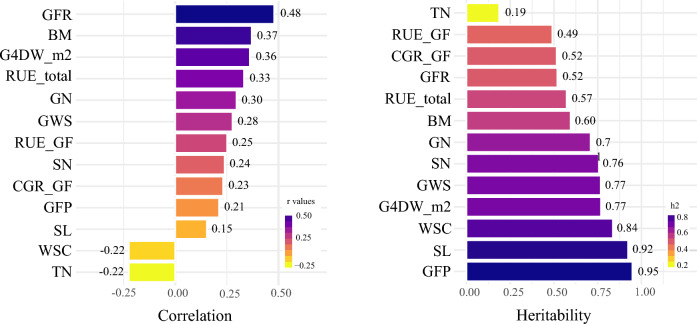
Traits significantly correlated with grain yield and their heritability.

Correlations were calculated to identify the GY relationship with agronomic and physiological traits. Significant Pearson’s coefficients (*r*) between GY and associated traits are given in Fig. 3. According to the *p* and* r* values, grain filling rate (GFR), biomass at crop maturity (BM), grain four weight per square meter (G4W_m^2^), radiation use efficiency calculated between 40 days after emergence and physiological maturity (RUE_total), grains number per m^2^ (GN) and grain weight per Spike (GWP) exhibited stronger and more significant positive correlations with GY, ranging from 0.28 to 0.48 (*p* < 0.001). Radiation use efficiency at grain filling (RUE_GF), spikes per square meter (SM2), crop growth rate at post-anthesis (CGR_GF), and grain filling period (GFP) had moderate associations with GY, exhibiting *r* values ranging from 0.21 to 0.25 (*p* < 0.01), while spike length (SL), water-soluble carbohydrates (WSC) and tillers per m^2^ (TN) showed the lowest significant correlation *r* values, ranging from 0.15 to − 0.22 (*p* < 0.05). The estimated heritabilities were moderate to high for most of the evaluated traits (Fig. 3), with GFP and SL exhibiting the highest values (0.95 and 0.92, respectively). Biomass at crop maturity, RUE_Total, and GWS displayed medium–high heritability (0.57 to 0.77), whereas TN exhibited the lowest heritability value (0.19).

### Stepwise regression and hierarchical clustering analysis

**Figure 4:**
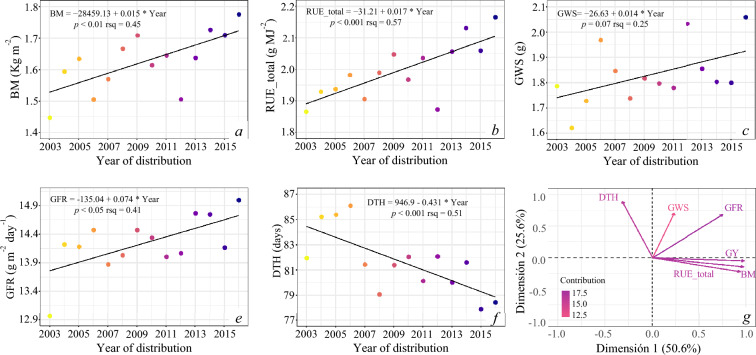
Associated traits genetic progress for top-performing CIMMYT’s International Elite Spring Wheat Yield Trials (ESWYT) genotypes selected from trials distributed from 2003 to 2016 (24th to 37th ESWYT) and phenotyped under optimal conditions at Norman E. Borlaug Experiment Station from 2018 to 2021. (**a**) *BM* biomass at maturity, (**b**) *RUE_total* total radiation use efficiency, (**c**) *GWS* grain weight per spike, (**d**) *GFR* grain filling rate, (**e**) *DHT* days to heading, and (**f**) principal component analysis of grain yield and the five related traits included in the stepwise regression model.

Since multicollinearity can increase the variances of the parameter estimates, thereby possibly causing insignificant predictors even though the overall model is significant, we calculated and examined the traits correlation matrix before running the stepwise regression model. The results obtained by using stepwise regression to select the best model from the predictors are given in Table 2. The subset of independent variables that best predicts the dependent variable GY included BM, GFR, DTH, GWS, and RUE_total. The individual trait contribution ranged from 0.1 to 73%, where the most significant traits were BM and GFR explaining 73 and 12% of the total contribution, respectively. According to the results, 96% of the total variations in grain yield were explained by the five selected variables. Analyzing the traits that significantly contributed to GY enhancement, with the exception of DTH, all of them showed positive trends over the years (Fig. 4). Overall BM ranged from 1.45 (24th ESWYT) to 1.78 (37th ESWYT) kg m^−2^, exhibiting a significant (*r* = 0.45; *p* < 0.01) annual progress rate of 1.03% (0.015 kg m^−2^ year^−1^). A similar trend was observed in RUE_total, which ranged from 1.86 (24th ESWYT) to 2.16 (37th ESWYT) g MJ^−1^ with a respective annual progress rate of 0.91% (0.017 g MJ^−1^ year^−1^) over the years (*r* = 0.57; *p* < 0.001). The regression line for GFR showed a significant genetic gain of 0.57% (0.074 g m^−1^ day^−1^) over the years of release (*r* = 0.41; *p* < 0.05). Grain weight per spike ranged from 1.62 (25th ESWYT) to 2.06 g (37th ESWYT) and although the trend was opposite for this trait (0.014 g year^−1^), no significant breeding effect was detected when they were regressed over ESWYT years (r = 0.25; *p* = 0.07). The DTH ranged from 77.9 (36th ESWYT) to 86.1 days (27th ESWYT) and showed a negative linear relationship of − 0.43 days year^−1^ (*r* = 0.51; *p* < 0.001) (Fig. 4f). All the traits included in the stepwise regression model showed a highly significant liner increases over the year, while DH was the only one where the quadratic model resulted significant as well (Table 1).

**Table 2:** Relative contribution (partial and model R^2^), AIC value, and residual sum of squares (RSS) in predicting wheat grain yield by the stepwise procedure analysis.

Variable	Step	Method	AIC	RSS	Sum sq	R^2^	Adj. R^2^	Partial R^2^
BM	1	Addition	15.30	3.97	10.99	0.73	0.72	0.73
GFR	2	Addition	− 20.09	2.13	12.83	0.86	0.85	0.12
DTH	3	Addition	− 68.96	0.91	14.05	0.94	0.93	0.08
GWS	4	Addition	− 81.38	0.72	14.24	0.95	0.95	0.01
RUE_total	5	Addition	− 89.10	0.61	14.35	0.96	0.95	0.01

**Figure 5:**
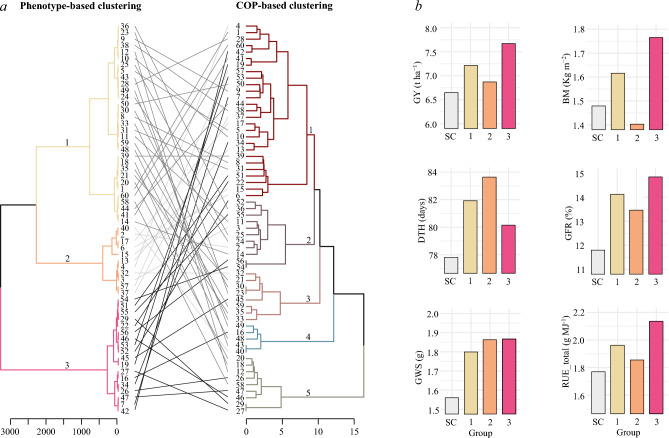
(**a**) Tanglegram comparison of ESWYT genotypes (n = 56) and four checks included in the study. Hierarchical clusters were performed based on the five traits included in the stepwise regression model (left) and coefficients of parentage (right) using the ward.D2 method (see codes of genotypes in Table S1). (**b**) Grain yield (GY), biomass at maturity (BM), days to heading (DTH), grain filling rate (GFR), grain weight per spike (GWS), and total radiation use efficiency (RUE_total) means performance of the groups identified in the phenotype-based clustering. *SC* check cultivar Siete Cerros.

The hierarchical clustering based on the traits significantly associated with GY in the stepwise regression model revealed three distinct groups of lines (Fig. 5a). The first group was formed by 31 lines, which included 28 genotypes and three checks (Baviacora M92, PBW343, and CIANO M2018). This group was comprised of lines from all nurseries, except the 37th ESWYT. On average, lines within group 1 exhibited intermediate GY, BM, RUE_total, GFR, and DTH values, and the lowest GWS values (Fig. 5b). Ten genotypes plus the check cultivar Siete Cerros were clustered in group 2, being most of the lines from the oldest nurseries. On average, they showed the lowest GY, BM, RUE_total, GFR, GWS values, and later DTH (Fig. 5b). Group 3 contained 18 genotypes, most of them from more recently distributed nurseries. As expected, this group was characterized by a better performance for most of the traits, including early heading (Fig. 5b). On the other hand, the hierarchical clustering analysis using the coefficient of parentage (COP) data allocated the lines in five different groups, revealing a different clustering pattern (Fig. 5a). In line with what was observed in the phenotypic grouping, no clear pattern was detected between the years of development and COP-based clustering. Through pedigree examination, group 1 (formed by the larger number of lines), did not show a clear prevalence of a particular parent, whereas groups 2, 3, 4, and 5 clustered genotypes were derived from Pastor, PBW343, Kachu, and Weebill#1, respectively. The tanglegram comparison demonstrated an important dissimilitude between phenotype and COP-based clustering with an entanglement value of 0.56, highlighting that both the positions and groupings of the genotypes were not consistent across the phenotypic and genotypic dendrograms.

## Discussion

Food demand is increasing worldwide, driven by population growth and dietary shifts. Simultaneously, the consequences of climate change pose a series of stresses on cropping systems that will severely endanger food production^3^. In this context, wheat breeding can significantly contribute to meeting future demand by increasing the rate of genetic gain, and therefore, assessing such a rate is critical. Based on the IWIN, which allows yearly evaluation of elite germplasm at several locations in different MEs or TPEs, and by including long-term checks in such trials, the CIMMYT Bread Wheat Breeding Program has been evaluating the grain yield genetic progress in different nurseries showing significant increases over time. Particularly for CIMMYT’s ESWYT germplasm, an early study analyzing 16th–30th ESWYTs nurseries in ME1 reported gains in the order of 27.4 kg ha^−1^ year^−1^ (0.55%) relative to the check genotype Atilla^10^, whereas, an analysis carried out on more recent nurseries (27th to 34th ESWYT), found grain yield gains relative to Atilla as 102.7 kg ha^−1^ year^−1^ (1.63%)^11^. Such differences in the rate of genetic gain may be a consequence of different evaluation periods (16th–30th vs. 27th to 34th ESWYT) as well as different statistical approaches for assessing the genotype × environment interaction^11^. However, the potential lack of uniform management across locations, differences in performance of used checks at each site, disease and pest control, as well as data quality of international trials data could also affect the results, representing a particular concern in such estimations^9^. For a more controlled genetic gains assessment, we evaluated a selection of high-performing internationally distributed ESWYT genotypes from 2003 (24th) to 2016 (37th) in common field trials under optimal conditions. Our set included genotypes from nurseries tested in both mentioned studies (25th to 34th ESWYT). Over 3 years of evaluations under optimally irrigated experiments, our results indicate a significant linear annual genetic gain of 65 kg ha^−1^ year^−1^ (0.96%). These results are intermediate to those reported in the two previous studies, supporting the validity of the estimates using international trial data as well as the significant progress made by the Bread Wheat Breeding Program over time. Our findings are also in agreement with Ref.^18^, who reported a genetic gain of 64 kg ha^−1^ year^−1^ (0.9%) for 26 spring wheat advanced lines developed by CIMMYT in the period from 1977 to 2008 and tested under optimally managed high-yielding environments. In addition to the increases in GY genetic gains, we were also able to demonstrate a positive trend in grain yield stability across experiments. More recently developed genotypes exhibited higher GY along with lower sensitivity and SRMSD, indicating that superior lines possess enhanced yield stability across multiple experiments, a key selection criteria by breeding programs to develop successful cultivars. Yield stability is a distinctive feature of CIMMYT material, achieved through the strategy of shuttle breeding (which aids the selection of photoperiod-insensitive and disease-resistant germplasm), together with the possibility of testing breeding material under different simulated growing conditions at the main yield testing site in Ciudad Obregon, Sonora, Mexico (Braun et al. 2010). The consistency observed between the genetic gain estimations derived from international data and the ERA trial data showcased in this study along with the observed genotype stability improvements (a key feature to face the climate change scenario) further underscores the Obregon station’s role as an exceptional breeding location for a vast global wheat cultivation area.

Evaluation of the agronomic and physiological traits relationships, as well as their relative contribution to grain yield is critical to identify yield-limiting factors and especially to plan future effective breeding strategies to steadily increase the grain yield by breeding programs. In our study, genetic grain yield progress under optimal conditions was shown to be related to increased BM, RUE_total, GFR, GWS at maturity, and reduced DTH. The total R^2^ explained by the stepwise regression analysis was 95.5%. The individual trait contribution ranged from 0.1 to 73%, being the most significant traits BM and GFR which explained 73 and 12% of the total grain yield variation, respectively. Individual regression analysis for the five traits included in the stepwise regression model indicated that RUE_total and BM have significantly increased over time, exhibiting rates of genetic gain of 0.017 g MJ^−1^ (*p* < 0.001) and 0.015 kg m^−2^ (*p* < 0.01), respectively. In line with these results, RUE_total and BM were also the strongest traits positively associated with GY (Fig. 4g). The contribution of biomass towards higher yield realization particularly in CIMMYT spring wheat germplasm has been reported in several studies^9,14^. The increase in biomass was mostly explained by increased RUE_total rather than increased cycle duration, which did not show a significant change over time (− 0.262 days year^−1^, *p* = 0.09). These findings demonstrate that, indirectly, breeders at CIMMYT have improved photosynthetic potential in spring bread wheat recently. In this line, it has been proposed that to further increase yield, it will be necessary to improve the RUE^26^, defined as the biomass accumulated per unit of absorbed radiation^27^. Different estimates from the literature show that the RUE of current wheat lines could be increased by up to 50% under favorable crop-growing conditions^23,24^.

Although only GFR showed a significant increase, both GFR and GWS had a positive contribution over the years (Fig. 4c,e). Increases in GWS were mainly associated with higher TKW (*r* = 0.68, p < 0.001) rather than grains per square meter, which exhibited a negative effect (*r* =  − 0.41, *p* < 0.001) (data not shown). Historical studies have shown that genetic progress in grain yield was mainly related to kernel number per unit of land area^17^, however, recent studies on CIMMYT germplasm suggest that grain yield genetic progress was mainly associated with grain weight^12,14,18^. The TKW increase in the CIMMYT spring wheat germplasm over the years could be associated with an increase in grain filling duration (significant reduction in DHT but not significant effect on PM) together with the visual selection of grain features such as grain size and plumpness carried out through different breeding generations. Significant associations between GY and grain weight under irrigated environments have also been reported by Ref.^9^. These authors found that the relative contribution of the GY component is related to the weather conditions during the crop season. Thus, while grain weight seems to be strongly associated with GY in optimal environments, under heat-stress environments the trait that tends to be associated with yield increase is grain number.

Grain yield performance is strongly influenced by the timing of the phenological stages in each environmental condition. Considering the wide variation of environments in CIMMYT target areas and in order to achieve a good adaptation, variations in genotype phenology are a critical component for the Bread Wheat Breeding Program at CIMMYT. In our study, we selected only four entries for each nursery/year based on their grain yield performance and stability across the international environments and although DTH (Fig. 4f) and PM revealed a negative trend over the years, there was significant variation within lines in each year. As mentioned before, increases in harvest biomass, the trait with the most important contribution to GY (73% of the total variation), were mostly explained by increased RUE_total rather than increased cycle duration. Total radiation use efficiency did not show a significant association with DH (*r* =  − 0.13, *p* = 0.16) or PM (*r* =  − 0.13, *p* = 0.15). These results suggest that further yield increases in cultivars adapted to different environments (different phenologies) could be achieved through BM and RUE_total improvement.

Secondary traits correlated with GY and easily measured in breeding programs can be used to predict the primary traits to improve genetic gain, particularly earlier in the breeding cycle before advancing to replicated yield trials^28^. In line with several previous studies, RUE_total and BM were traits significantly associated with GY improvement over time, supporting the value of including them in the breeding process. However, due to the complexity, destructive as well as labor, and cost-intensive determination, their potential for increasing yield has not been widely exploited in wheat breeding programs^29^. The advent of low-cost, non-invasive high-throughput phenotyping (HTP) technologies opens an opportunity to include such traits within the selection criteria. Recent studies using remote sensing models have been able to predict traits such as intercepted photosynthetically active radiation, RUE, and BM with up to 70% accuracy compared with ground truth data^29,30^. The indirect selection for such associated complex traits using HTP can provide the opportunity to introduce new alleles from which genetic progress can be made as well as increase selection intensity through the screening of larger populations.

The hierarchical cluster based on the five traits included in the stepwise model identified three distinct clusters (Fig. 5a). In the high-yielding cluster (group 3) several new lines were included and on average, they exhibited higher BM, RUE_total, and reduced DTH. However, the individual analysis of the top ten higher-yielding lines revealed that they differ for the mentioned traits indicating that no single traits criterion is adopted by CIMMYT breeders for developing new superior lines. Since the relative contribution of associated traits to GY has been reported to be different depending on the environment and growing conditions, the development of higher-yielding lines based on different trait combinations is essential to achieve adaptation to diverse environments^9^.

The grouping based on the genetic relationship (COP) of the lines was not consistent with the phenotypic-based clustering results, revealing five different groups (Fig. 5a). The entanglement value was 0.56 showing the divergence in genotype distribution in both dendrograms. Entanglement measures genotype matching between distinct dendrograms, ranging from 1 (complete entanglement) to 0 (no entanglement). A lower entanglement coefficient corresponds to a good alignment^31^. Weak or no correlations between phenotypic and genetic data in wheat have been found previously^32^. Based on the pedigree examination, it was possible to determine that lines clustered in specific groups (using the COP) are derived from cultivars such as Pastor (group 2), PBW343 (group 3), Kachu (group 4) and Weebill#1 (group 5). These genotypes and their derivatives have been released and grown extensively by farmers as well as utilized as parents in breeding programs due to their superior and stable performance over a range of different environments^33^. Contrary, higher-yield lines did not show a clear grouping pattern based on COP, indicating the existence of genetic variability among them. Based on these results, future yield increases could be achieved by crossing such lines and selecting superior recombinants.

In conclusion, our study demonstrated significant linear and continued grain yield progress together with enhanced stability in ESWYT germplasm developed and delivered by the CIMMYT’s Bread Wheat Breeding Program, without signs of stagnation. The observed rate of genetic gain was comparable to previous studies using international trial data, which supports the validity of such analyses in estimating genetic progress from the existing data to assess the performance of breeding programs. The dissection of several agronomic and physiological features revealed some promising traits such as RUE_total contributing significantly to grain yield. With the advent of technologies like HTP, such traits could be used in the early selection of materials to further increase genetic gains. Finally, the differential contributions of GY-associated traits in the top ten highest-yielding lines demonstrated that improved grain yield can be achieved through different mechanisms and indicated that no single trait criterion is adopted by CIMMYT breeders for developing new superior lines.

## Materials and methods

### Plant material

The experimental material consisted of 60 spring bread wheat genotypes, including four checks (Siete Cerros, Baviacora M92, PBW343, and Ciano M2018) and 56 elite lines from the international 24th–37th Elite Spring Wheat Yield Trial (ESWYT) from 2003 to 2016 (Table S1). The best four internationally performing lines from each of the fourteen ESWYTs were selected based on their GY performance and stability across international sites in over 70 countries.

### Field trials, experimental design, and trait determination

The study was conducted at the Norman E. Borlaug Experiment Station, Ciudad Obregon (27° 20′ N, 109° 54′ W), Sonora, Mexico. Trials were planted on: (1) Beds with five irrigations (B5IR): genotypes were grown on raised beds with about 500 mm of available water and optimal sowing date during late November–early December, and (2) Flat with five irrigations (F5IR): genotypes were grown on a flat field with about 500 mm of available water and optimal sowing date. For both planting systems, five irrigations of approximately 100 mm each and equally distributed across the growing season were applied through drip systems. At sowing, ∼ 100 kg ha^−1^ N (granulated urea) and 25 kg ha^−1^ P (calcium triple superphosphate) were applied across both environments, which was followed by another N application around the first node initiation of 120 kg ha^−1^ (granulated urea). Folicur and Admire were applied at recommended rates twice during the growing season to protect against rust diseases and aphids, respectively. There were two trials grown under flat planting; one of these was used to conduct both yield and yield component and physiological measurements (e.g. biomass, radiation use efficiency, etc.) whereas the other was used to quantify only yield.

All trials were arranged in an alpha lattice design with three replicates and a 6.1 m^2^ plot size with a seed rate of 120 kg ha^−1^. The trials were conducted during three consecutive growing seasons 2018–2019 (18–19), 2019–2020 (19–20), and 2020–2021 (20–21), resulting in a total of nine experiments. In addition to grain yield, 35 agronomic and physiological features were measured. A complete trait description is provided in Supplementary Table S2 (Additional information can also be found at https://repository.cimmyt.org/handle/10883/1288).

### Data analysis

The phenotypic data were analyzed using the *lme4* package^34^ in R software^35^. First, the GY of each trial was analyzed individually to estimate heritability (*H*^2^) using the following model:

$${\mathbf{Y}}_{{{\text{ijk}}}} = \mu + {\text{ R}}_{{\text{j}}} + {\text{ B}}_{{\text{k}}} \left( {{\text{R}}_{{\text{j}}} } \right) \, + {\text{ G}}_{{\text{i}}} + \, \varepsilon_{{{\text{ijk}}}} ,$$

where *μ* is the general mean, R_j_ is the random effects of the replicates (j = 1,…,3), G_i_ is the random effects of the wheat genotypes (i = 1,…, 60), assumed to be identically and independently normally distributed (iid) with mean zero and variance σ2g, and B_k_ represents the random effects of the incomplete blocks (k = 1,…, 5), assumed (iid) with mean zero and variance σ^2^_sb(r)_. The term ε_ijk_ is a random residual assumed to be iid with mean zero and variance σ^2^_ε_. Then, we fit the same model but now with G_i_ as fixed effects to estimate adjusted means (Best Linear Unbiased Estimates, BLUE) and calculated Pearson correlations between trials using the R package *GGally*^36^. Based on GY data, trials not significantly correlated or with H^2^ < 0.15 were excluded from further analysis.

The combined analyses across environments were performed using the following linear mixed model:

$${\mathbf{Y}} = {\mathbf{1}}\mu + {\mathbf{X}}_{{\mathbf{s}}} {\mathbf{s}} \, + \, {\mathbf{Z}}_{{\mathbf{r}}} {\mathbf{r}} \, + \, {\mathbf{Z}}_{{\mathbf{b}}} {\mathbf{b}} \, + \, {\mathbf{Z}}_{{\mathbf{g}}} {\mathbf{g}} \, + \, {\mathbf{Z}}_{{{\mathbf{ge}}}} {\mathbf{ge}} + {\mathbf{e}},$$

where **1** is a column vector of ones, *μ* is the overall mean, **X**_**s**_ is the incidence matrix for the fixed effects of environment, and **Z**_**r**_**, Z**_**b**_**, Z**_**g**_, and **Z**_**ge**_ are the design matrices for the random effects of replicates within environments, incomplete blocks within replicates and environment, genotypes, and genotype × environment (GE), respectively. Vectors denote the fixed effect of environments; while vectors **r, b, g, ge,** and **e** contain random effects of replicates within environments and incomplete blocks within replicates and environments, genotypes, GE, and residuals, respectively. These are assumed to be iid random variables, normally distributed with zero mean vectors and variance–covariance matrices R, B, G, GE, and E, respectively.

### Computing genetic gains

We followed the procedures described in Excellence in Breeding (EiB) breeding scheme optimization manuals (https://excellenceinbreeding.org/toolbox/tools/eib-breeding-scheme-optimization-manuals). Briefly, the adjusted GY means were regressed over the years of ESWYT distribution, and the realized rate of genetic gain was calculated using the slope of the regression line. The genetic gain was also calculated in terms of the check average, where the differences in percentage were also regressed over the years of ESWYT evaluation. In order to assess linearity versus non-linearity trait response, orthogonal polynomials were used to fit different orders of polynomials (e.g., linear, quadratic) to determine which model best describes the relationship on trait response. In addition to the genetic gains estimations, genotype stability was evaluated according to Ref.^37^ using in R package *statgenGxE*^38^ the model below:

$${\mathbf{Y}}_{ij} = {{\varvec{\upmu}}} + {\mathbf{G}}_{i} + {{\varvec{\upbeta}}}_{i} {\mathbf{E}}_{j} + {{\varvec{\upepsilon}}}_{ij} ,$$

where Y_*ij*_ is the phenotypic value of genotype i in environment j, μ is the general mean, G_*i*_ is the genotypic effect, β_*i*_ a sensitivity parameters, E_*j*_ the environment effect and ϵ_*ij*_ a residual.

Trials-adjusted means were used to estimate the Pearson correlations among traits. A stepwise regression analysis was performed to further identify the key traits responding to yield realization and their relative contribution using the R packages *MASS* and *olsrr*^39,40^. Before running the step-wise regression models, *findLinearCombos* and *findCorrelation* functions from the R package *caret* were applied to the complete data set to reduce the dimensionality and remove highly correlated traits (cutoff = 0.65) which may reduce the effectiveness of regression models^41^. Finally, hierarchical clusters based on significant traits included in the stepwise model as well as the coefficients of parentage (COP) of the lines were generated using the multivariate data analyses in R package *FactoMineR*^42^. The cluster solution (k) was equal to the number of groups identified in the PCA analysis. Phenotype and COP-based clustering were compared using the R package *dendextend*^43^ to observe grouping patterns between phenotypic and genotypic (pedigree) data.

### Weather conditions

Considering the whole growing season (November to April), trends in mean and maximum temperatures as well as in precipitation were similar across the trial years. Mean and maximum temperatures ranged from 20.19 to 20.69 and 33.5 to 35.7 °C in the 18–19 and 20–21 crop seasons, respectively. While the total precipitation was 1.74, 3.84, and 0.69 mm in the 18–19, 19–20, and 20–21 crop seasons, respectively.

### Ethics approval

We confirmed that all methods used in this study were carried out in accordance with relevant guidelines in the “Materials and methods” section.

### Supplementary Information

Supplementary Table S1.

Supplementary Table S2.

Supplementary Table S3.

## Data Availability

The datasets generated and analyzed during the current study are available in the CIMMYT Dataverse repository: https://hdl.handle.net/11529/10548957.
