# Author Correction: Enhanced radiation use efficiency and grain filling rate as the main drivers of grain yield genetic gains in the CIMMYT elite spring wheat yield trial

**DOI:** 10.1038/s41598-024-67938-2

**Published:** 2024-07-30

**Authors:** Guillermo Gerard, Suchismita Mondal, Francisco Piñera‑Chávez, Carolina Rivera‑Amado, Gemma Molero, Jose Crossa, Julio Huerta‑Espino, Govindan Velu, Hans Braun, Ravi Singh, Leonardo Crespo‑Herrera

**Affiliations:** 1 International Maize and Wheat Improvement Center (CIMMYT), Carretera México- Veracruz Km. 45, El Batán, 56237 Texcoco, Mexico, CP Mexico; 2 Plant Sciences and Plant Pathology Department, Montana State University, Bozeman, MT 59717 USA; 3 KWS Momont Recherche, 59246 Mons-en-Pévèle, Hauts-de-France, France; 4 Colegio de Postgraduados, 56230 Montecillos, Mexico, CP Mexico; 5 Campo ExperimentalValle de Mexico-INIFAP, Texcoco, Mexico, Mexico

Correction to: *Scientific Reports* 10.1038/s41598-024-60853-6, published online 14 May 2024

The original version of this Article contained an error in the spelling of the author Govindan Velu which was incorrectly given as Govidan Velu.

The original Article has been corrected.

